# scRNA-seq Profiling of Human Testes Reveals the Presence of the ACE2 Receptor, A Target for SARS-CoV-2 Infection in Spermatogonia, Leydig and Sertoli Cells

**DOI:** 10.3390/cells9040920

**Published:** 2020-04-09

**Authors:** Zhengpin Wang, Xiaojiang Xu

**Affiliations:** 1 Laboratory of Cellular and Developmental Biology, NIDDK, National Institutes of Health, Bethesda, MD 20892, USA; zhengpin.wang@nih.gov; 2 Integrative Bioinformatics, ESCBL, NIEHS, National Institutes of Health, Research Triangle Park, NC 27709, USA

**Keywords:** SARS-CoV-2, infection, scRNA-seq, ACE2, spermatogonia

## Abstract

In December 2019, a novel coronavirus (SARS-CoV-2) was identified in COVID-19 patients in Wuhan, Hubei Province, China. SARS-CoV-2 shares both high sequence similarity and the use of the same cell entry receptor, angiotensin-converting enzyme 2 (ACE2), with severe acute respiratory syndrome coronavirus (SARS-CoV). Several studies have provided bioinformatic evidence of potential routes of SARS-CoV-2 infection in respiratory, cardiovascular, digestive and urinary systems. However, whether the reproductive system is a potential target of SARS-CoV-2 infection has not yet been determined. Here, we investigate the expression pattern of ACE2 in adult human testes at the level of single-cell transcriptomes. The results indicate that ACE2 is predominantly enriched in spermatogonia and Leydig and Sertoli cells. Gene Set Enrichment Analysis (GSEA) indicates that Gene Ontology (GO) categories associated with viral reproduction and transmission are highly enriched in ACE2-positive spermatogonia, while male gamete generation related terms are downregulated. Cell–cell junction and immunity-related GO terms are increased in ACE2-positive Leydig and Sertoli cells, but mitochondria and reproduction-related GO terms are decreased. These findings provide evidence that the human testis is a potential target of SARS-CoV-2 infection, which may have significant impact on our understanding of the pathophysiology of this rapidly spreading disease.

## 1. Introduction

In December 2019, a novel coronavirus designated SARS-CoV-2 was identified in patients with pneumonia in Wuhan, Hubei Province of China. The new coronavirus disease has received an official name, coronavirus disease 2019 (COVID-19) from the World Health Organization. It can cause acute respiratory distress syndrome and infected patients have a relatively high risk of death [1,2,3]. It has been reported that SARS-CoV-2 shares 76% amino acid sequence identity with severe acute respiratory syndrome coronavirus (SARS-CoV), and is likely to use the same receptor, angiotensin-converting enzyme 2 (ACE2), for entry into target host cells [4,5,6]. Recent studies provide structural and functional evidence that SARS-CoV-2 entry into cells is recognized by full-length human ACE2 [7,8]. Another experimental study reveals that SARS-CoV-2 employs the SARS-CoV receptor ACE2 for host cell entry [5]. Collectively, structural and functional studies have demonstrated that SARS-CoV-2 uses the SARS-CoV receptor ACE2 for entry.

scRNA-seq analysis documents that ACE2 is specifically expressed in type II alveolar epithelial cells (AT2) in human lungs [6], suggesting that this virus targets ACE2-positive AT2 cells to induce pneumonia. Liver function damage has been reported in SARS-infected and Middle East respiratory syndrome coronavirus (MERS-CoV)-infected patients [9,10]. A recent epidemiologic study indicates that some patients infected with SARS-CoV-2 have signs of severe liver damage [1]. By analyzing healthy liver cells at single-cell resolution, investigators have determined that ACE2 is significantly enriched in cholangiocytes [11], suggesting that the virus might directly bind to ACE2-positive cholangiocytes to dysregulate liver function. Moreover, a recent study explored the composition and proportion of ACE2-expressing cells in the digestive system by scRNA-seq analysis, and its results showed that ACE2 is not only highly expressed in lung AT2 cells, the upper esophagus and stratified epithelial cells, but also in absorptive enterocytes in the ileum and colon [12], implying that the digestive system is a potential route of SARS-CoV-2 infection. In addition, cardiovascular and urinary systems have been reported as potential organ targets of SARS-CoV-2 infection [13].

A series of studies have provided the bioinformatics evidence of potential routes of SARS-CoV-2 infection in respiratory, cardiovascular, digestive and urinary systems. SARS-infected male patients show wide-spread germ cell destruction, few or no spermatozoon in the seminiferous tubules, and a thickened basement membrane in the testes [14]. However, whether the reproductive system is susceptible to SARS-CoV-2 infection has not yet been determined. In this study, we investigate the RNA expression profiles of ACE2 in adult human testes at single-cell resolution. Our study documents that ACE2 is predominantly enriched in spermatogonia and Leydig and Sertoli cells. ACE2-positive cells possess higher abundance of transcripts associated with viral reproduction and transmission, and lower abundance of transcripts related to male gametogenesis. Thus, the reported ACE2 expression in human testes suggests that SARS-CoV-2 could infect the male gonad and risk male reproductive dysfunction.

## 2. Materials and Methods

### 2.1. Data Sources

Adult human testis scRNA-seq datasets were obtained from Gene Expression Omnibus (GEO) and Sequence Read Archive (SRA) databases under the accession number GSE109037.

### 2.2. scRNA-Seq Data Processing

Raw read processing was carried out using the Cell Ranger Single-Cell Software Suite (version 3.1.0, 10X Genomics Inc., CA, USA). The primary data analyses which included alignment, filtering, barcode counting and unique molecular identifier (UMI) quantification for determining gene transcript counts per cell (generating a gene-barcode matrix) and quality control, were performed using the Cell Ranger *count* command. Gene positions were annotated using Ensembl build 93 and were filtered for biotype (protein-coding, long intergenic noncoding RNA, antisense, immunoglobulins and T-cell receptors only).

### 2.3. Single-Cell Transcriptomes to Identify Cell Types

Raw gene expression matrices generated per sample using Cell Ranger (Version 3.1.0) were imported into R (Version 3.6.2) and converted into a Seurat object using the Seurat R package (Version 3.1.2). Cells which had either fewer than 300 expressed genes or over 15% UMIs derived from the mitochondrial genome were discarded. For the remaining cells, gene expression matrices were normalized to total cellular read count and to mitochondrial read count using the negative binomial regression method implemented in the Seurat *SCTransform* function. Cell-cycle scores were also calculated using the Seurat *CellCycleScoring* function since the cell cycle phase effect was observed. The gene expression matrices were then further normalized to cell cycle scores. The Seurat *RunPCA* functions were used to calculate the principal components (PCs). We further performed the batch effect correction using Harmony, because batch effects among the three human testis samples were observed. The *RunUMAP* function in its default setting was applied to visualize the first 35 Harmony-aligned coordinates. The *FindClusters* function with a resolution = 0.6 parameter was carried out in order to cluster cells into different groups. Canonical marker genes were applied to annotate cell clusters into known biological cell types.

### 2.4. Identification of Differential Expression Genes 

To identify differential expression genes (DEG) between two groups, we used the Seurat *FindMarkers* function with the default parameter of the “MAST” method and cell IDs from each defined group (e.g., AT2 with ACE2 expression vs. AT2 without ACE2 expression) as inputs.

### 2.5. Gene Function Analysis

Gene Set Enrichment Analysis (GSEA, Version 4.3) was used to complete Gene Ontology (GO) term enrichment analysis with the Molecular Signatures Database (MSigDB) C5 GO gene sets (Version 7.0).

## 3. Results

### 3.1. Identification of Cell Types in Adult Human Testes

To assess the expression pattern of ACE2 in human testes, we first analyzed a published scRNA-seq dataset from three individual adult human testis samples [15]. From a total of 17,520 testicular cells, 16,632 cells passed standard quality control and were retained for subsequent analyses. On average, we detected 9398 UMIs and 2388 genes in each individual cell.

**Figure 1:**
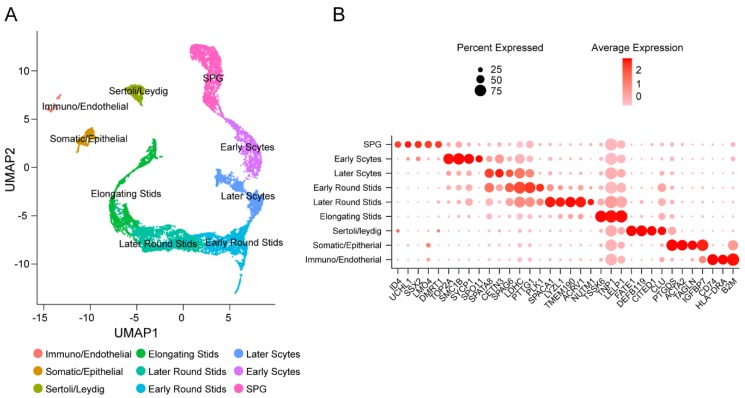
Single-cell transcriptome profiling from published adult human testes. (**A**) Uniform manifold approximation and projection (UMAP) clustering of combined adult human testicular cells from three individual samples. Nine major cell clusters were identified across a total of 16,632 cells. (**B**) Dot plot of proportion of cells in the respective cluster expressing selected marker genes (dot size), and average expression (color scale). SPG, spermatogonia; Early S’cytes, early spermatocytes; Late S’cytes, late spermatocytes; Early Round S’tids, early round spermatids; Later Round S’tids, later round spermatids; Elongating S’tids, elongating spermatids; Immuno, immune cells.

Uniform manifold approximation and projection (UMAP) and marker gene analyses were performed for cell type identification of the total 16,632 testicular cells. Based on the UMAP results, we identified nine major cell clusters, and none of the clusters solely derived from one individual, as shown in Figure 1A,B. Cluster identity was assigned based on expression patterns of known marker genes in human testes. We have identified five major germ cell types including spermatogonia, early spermatocytes, late spermatocytes, round spermatids and elongated spermatids that recapitulated the temporal order of spermatogenesis. We also identified somatic cell types including endothelial, Sertoli and Leydig cells as well as monocytes, as shown in Figure 1A,B.

### 3.2. Cell-Specific Expression of ACE2

**Figure 2:**
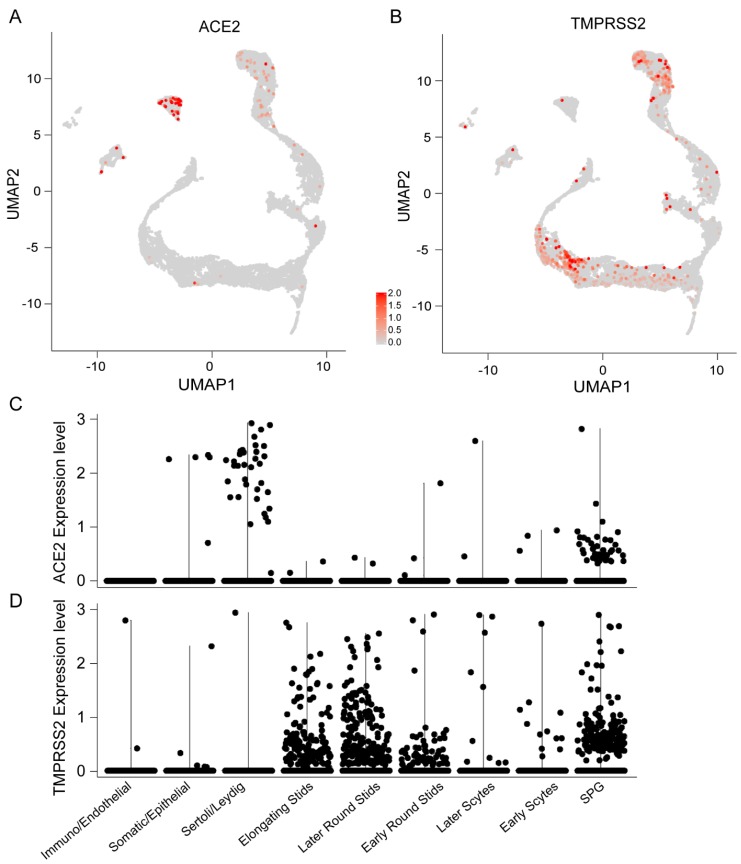
Angiotensin-converting enzyme 2 (ACE2) expression pattern in adult human testes. (**A**) Per-cell expression level of ACE2 of human testicular cells visualized on the UMAP plot. (**B**) UMAP plot of transmembrane serine protease 2 (TMPRSS2) expression across all cell clusters. (**C**) Violin plots of ACE2 expression in all identified cell types. (**D**) Violin plots of TMPRSS2 expression across all cell types. SPG—spermatogonia.

To determine the specific cell types expressing ACE2, we analyzed the RNA expression profile of ACE2 at single-cell resolution in human testes. Since we could not separate Leydig and Sertoli cells as distinct clusters, we combined these two somatic cell types together for subsequent analyses. The UMAP plot revealed that ACE2 was primarily enriched in two major clusters corresponding to spermatogonia and Leydig and Sertoli cells, as displayed in Figure 2A. A violin plot further demonstrated that ACE2 was highly expressed in spermatogonia and Leydig and Sertoli cells. Early spermatocytes, late spermatocytes, spermatids and other somatic cells had very low expression levels of ACE2, as displayed in Figure 2C. A recent study reported that SARS-CoV-2 uses the SARS-CoV receptor ACE2 for host cell entry, and the transmembrane serine protease 2 (TMPRSS2) for viral spike (S) protein priming [5]. Feature and violin plots indicated that TMPRSS2 expression was concentrated in spermatogonia and spermatids with relatively low levels in other cell types, as shown in Figure 2B,D. Thus, TMPRSS2 expression in spermatogonia and ACE2 expression in spermatogonia and Leydig and Sertoli cells suggest a high potential of SARS-CoV-2 infection in human testes.

**Figure 3:**
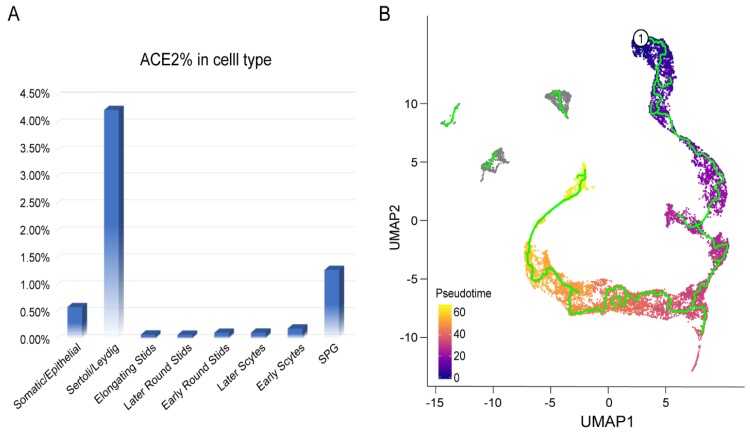
Composition of ACE2-positive cells and pseudotime analysis of human testicular cells. (**A**) ACE2-expression cells in each identified cell type. SPG—spermatogonia. (**B**) Trajectory of male germ cell development by pseudotime time analysis of human testicular cells.

We further analyzed the proportion of ACE2-positive cells of human testicular cells. We found that ACE2-positive spermatogonia represented 1.28% of all spermatogonia in human testis as shown in Figure 3A, with similar expression level of ACE2-expressing cells (1.40% ± 0.40%) in AT2 cells [6]. The enrichment of ACE2 in Leydig and Sertoli cells had a 3-fold higher percentage when compared with ACE2-expressing cells in AT2 cells (4.25% versus 1.40%), as displayed in Figure 3A. In addition, pseudotime analysis provided the trajectory of male germ cell development as shown in Figure 3B, and further suggested that spermatogenesis would be disrupted if spermatogonia were infected and damaged by SARS-CoV-2.

### 3.3. Characteristics of ACE2-Positive Cells in Human Testis

**Figure 4:**
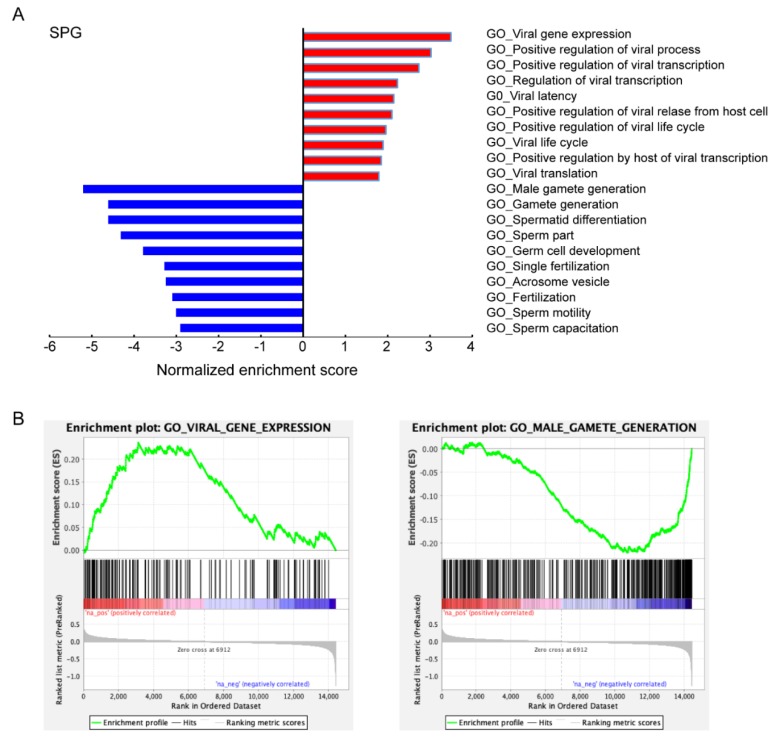
Characteristics of ACE2-positive spermatogonia. (**A**) Gene ontology enrichment analysis of biological process categories of ACE2-positive spermatogonia compared with ACE2-negative spermatogonia. (**B**) Examples of the enrichment plot for terms of viral gene expression and male gamete generation.

In order to further characterize ACE2-positive cells in human testes, GSEA was performed by comparing ACE2-positive cells with ACE2-negative cells to determine which biological processes were enriched within either spermatogonia or Leydig and Sertoli cells. We found that 24 GO terms associated with viral reproduction and transmission were positively enriched in ACE2-positive spermatogonia and included viral gene expression (e.g., NUP133, POLR2A, JUN, RANBP2, RPL12, EIF3L, RPL3, RPL4, RPS19, RPS2, NUP85), the positive regulation of viral processes (e.g., TOP2A, RSF1, PPIA, CHMP2A, NUCKS1, TRIM11, POLR2B, NELFB, CHD1, NELFCD, TSG101), viral latency, the positive regulation of viral release from host cells, viral life cycle, viral translation, viral genome replication and viral budding, as displayed in Figure 4A,B. In contrast, multiple GO terms related to male reproduction were significantly decreased in ACE2-positive spermatogonia. These GO terms included male gamete generation (e.g., ADCY10, METTL3, RNF8, CDC42, SYCP1, DAZL, ETV5, YTHDC2, TEX14, REC8, TAF7L, YBX2, NANOS3, DDX4, SYCE3), spermatid differentiation (e.g., SPAG16, CFAP157, OCA2, RFX2, PYGO1, TTC26), fertilization (e.g., NECTIN2, PLB1, CCT7, NPM2, IZUMO1, SPAG8, CD9), sperm motility (e.g., SORD, ANXA5, SLC22A16, CFAP44, SLIRP), sperm capacitation (e.g., PEBP1, CATSPERD, SLC26A6, CATSPER3), sperm-egg recognition, acrosome reaction, sperm chromatin condensation and male meiosis, as shown in Figure 4A,B. The GSEA results suggest that male germ cell specific genes and genes that are collectively involved in spermatogenesis are compromised in ACE2-positive cells. Therefore, SARS-CoV-2 may directly target ACE2-positive spermatogonia and disrupt spermatogenesis.

**Figure 5:**
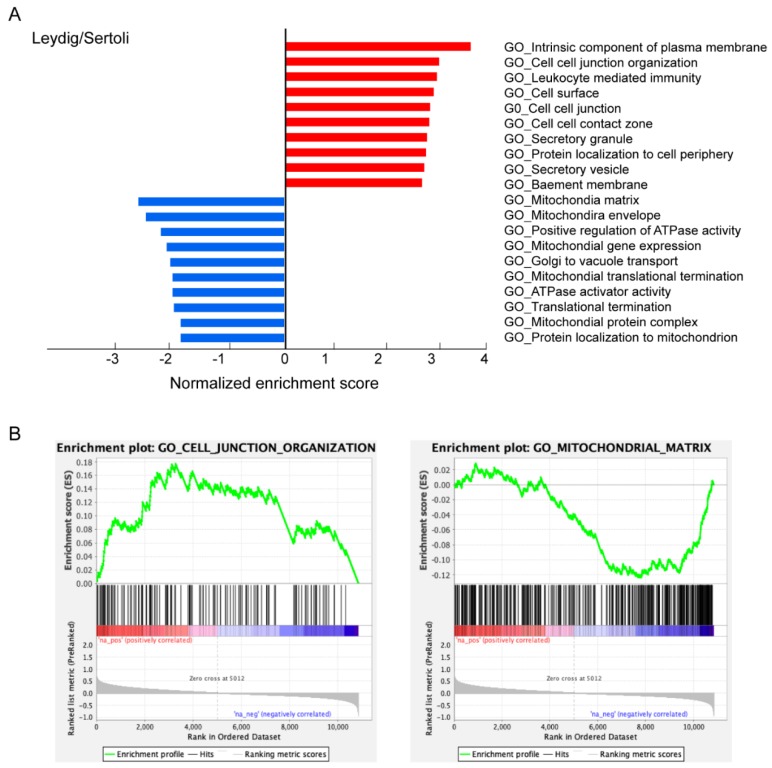
Characteristics of ACE2-positive Leydig and Sertoli cells. (**A**) Gene ontology enrichment analysis of biological process categories of ACE2-positive Leydig and Sertoli cells compared with ACE2-negative cells. (**B**) Examples of the enrichment plot for terms of cell–cell junction organization and mitochondrial matrix.

We further compared the characteristics of ACE2-positive Leydig and Sertoli cells with ACE2-negative cells. The GSEA documented that cell junction and immunity related GO terms were enriched in ACE2-positive cells, including cell–cell junction organization (e.g., *ACE2*, *FLCN*, *WHRN*, *MTDH*, *RHOA*, *CTNNA1*), leukocyte mediated immunity (e.g., *MLEC*, *KPNB1*, *SPTAN1*, *PRSS3*, *PAFAH1B2*, *KCMF1*), cell surface (e.g., *CTSV*, *SPARC*, *PTN*, *HSPA5*, *PHB2*, *APP*), the cell–cell contact zone (e.g., *CXADR*, *CTNNB1*, *CDH2*, *PCDH9*, *DLG1*, *GJA1*), secretory granules, cell activation, the immune effector process and exocytosis, as shown in Figure 5A,B. Thus, SARS-CoV-2 may replicate and transfer through cell–cell junctions. Contrarily, some mitochondria and reproduction related GO terms were decreased in ACE2-positive cells, including mitochondrial matrix, the mitochondrial envelope, mitochondrial gene expression (e.g., *MRPS18A*, *MRPL21*, *QRSL1*, *MTERF2*, *COA3*, *MRPL58*, *HARS*, *MTOL*, *RCC1L*), mitochondrial translational termination, ATPase activator activity, fertilization, spermatid differentiation, sperm capacitation, sperm motility and sperm egg recognition, also displayed in Figure 5A,B. These data suggest that ACE2-positive Leydig and Sertoli cells have lower potential to support spermatogenesis.

## 4. Discussion

Two known coronaviruses, SARS-CoV and MERS-CoV, are infection sources of respiratory disease in humans that caused public panic in past years [16]. In December 2019, a newly identified coronavirus (SARS-CoV-2) was discovered in patients that had similar respiratory symptoms with SARS and MERS. Until now, no effective drugs are clinically approved for these etiologic agents, but it appears that they share the same ACE2 receptor for entry into the host cells for reproduction and transmission [5,7,8]. Thus, the investigation of the composition and expression pattern of ACE2 may suggest potential routes of SARS-CoV-2 infection in humans. Recent studies have shown that respiratory, cardiovascular, digestive and urinary systems are affected by SARS-CoV-2 infection [12,13]. Based on our current study of the scRNA-seq data in adult human testes, we suggest that the testis is also potentially vulnerable to SARS-CoV-2 infection.

Mammalian spermatogenesis is a coordinate and dynamic cell differentiation process supported by the self-renewal and differentiation of spermatogonial stem cells (SSCs). It is stringently controlled in a special niche microenvironment in testicular seminiferous tubules. Sertoli cells are the only somatic cell type in the tubules and directly interact with spermatogenic cells to control spermatogenic cell differentiation through paracrine signaling [17]. The interstitial cells of Leydig are adjacent to the seminiferous tubules and produce testosterone in the presence of luteinizing hormones to support spermatogenic cell differentiation [18]. Functional abnormalities in male germ cells or these supporting somatic cells cause spermatogenic failure and male infertility.

SARS coronavirus has been known to damage multiple organs including human testes. SARS can cause orchitis in humans. SARS-infected patients show testis damage and defects in spermatogenesis [14]. All SARS testes display wide-spread germ cell destruction, few or no spermatozoon in the seminiferous tubules and leukocyte infiltration [14]. SARS-CoV-2 sharing the same receptor as SARS raises the possibility that the human testis is also a potential route of SARS-CoV-2 infection.

By analyzing the expression pattern of ACE2 in adult human testes at single-cell transcriptome resolution, we found that ACE2 is primarily expressed in spermatogonia and Leydig and Sertoli cells in the human testis. ACE2-positive spermatogonia express a higher number of genes associated with viral reproduction and transmission, and a lower number of genes related to spermatogenesis compared to ACE2-negative spermatogonia. ACE2-positive Leydig and Sertoli cells express higher genes involved in cell–cell junction and immunity, and lower genes associated with mitochondria and reproduction. These findings suggest that the testis is a high-risk organ vulnerable to SARS-CoV-2 infection that may result in spermatogenic failure. 

In summary, our study provides bioinformatics evidence that the testis may be potentially vulnerable to SARS-CoV-2 infection. These investigations suggest that the reproductive functions should be followed and evaluated in recovered COVID-2019 male patients. Our findings may also have translational implications for the treatment of reproductive defects caused by SARS-CoV-2 infection.
